# ASSOCIATION OF DIABETES AND VAGINAL MICROBIOTA WITH THE GENITOURINARY SYNDROME OF MENOPAUSE

**DOI:** 10.1093/geroni/igad104.1440

**Published:** 2023-12-21

**Authors:** Sarah Robbins, Michelle Shardell, Amber Beitelshees, Ryan Miller, Jacques Ravel, Simeon Taylor, Rebecca Brotman

**Affiliations:** University of Maryland School of Medicine, Baltimore, Maryland, United States; University of Maryland School of Medicine, Baltimore, Maryland, United States; University of Maryland, Baltimore, Baltimore, Maryland, United States; University of Maryland School of Medicine, Baltimore, Maryland, United States; University of Maryland School of Medicine, Baltimore, Maryland, United States; University of Maryland School of Medicine, Baltimore, Maryland, United States; University of Maryland School of Medicine, Baltimore, Maryland, United States

## Abstract

The genitourinary syndrome of menopause (GSM) comprises urogenital signs and symptoms associated with estrogen deficiency during menopause. For many, decreased estrogen reduces vaginal Lactobacillus species abundance, a state associated with adverse urogenital outcomes. For these reasons, vaginal microbiota in women’s reproductive aging is an emerging topic of interest in geroscience research. The risk of diabetes also increases with menopause; however, few studies have jointly assessed diabetes and vaginal microbiota with GSM. Therefore, we assessed the relationships between diabetes, vaginal microbiota and GSM in a study of 811 participants aged 35-60 recruited to a 2-year observational cohort with follow-up every 6 months. Diabetes was defined as self-reported diabetes medications, history, or serum glycated albumin >16.5%. There were 22 and 251 postmenopausal participants with and without diabetes, respectively. Mixed-effects logistic regression quantified the association of vaginal microbiota with GSM outcomes among those with and without diabetes. After adjustment for covariates, low-Lactobacillus vaginal microbiota states were associated with lower odds of vulvovaginal atrophy (VVA) (aOR=0.23, 95% CI: 0.04-1.33) among postmenopausal participants with diabetes; however, this finding was not statistically significant. In contrast, among postmenopausal participants without diabetes, low-Lactobacillus microbiota was significantly associated with higher odds of VVA (aOR=1.63, 95% CI: 1.07-2.47). Diabetes significantly modified the association between vaginal microbiota and VVA among postmenopausal individuals (p=.032). Findings were similar for clinician-assessed vaginal dryness. These results suggest that the relationship between vaginal microbiota and GSM may differ by diabetes status. Larger studies are needed to further understand the relationships between diabetes, vaginal microbiota and GSM.

